# Is Handedness at Five Associated with Prenatal Factors?

**DOI:** 10.3390/ijerph18073529

**Published:** 2021-03-29

**Authors:** Jacqueline Fagard, Maria De Agostini, Viviane Huet, Lionel Granjon, Barbara Heude

**Affiliations:** 1 Université de Paris, INCC UMR 8002, CNRS, F-75006 Paris, France; viviane.huet@u-paris.fr (V.H.); lionel.granjon.lpp@gmail.com (L.G.); 2 Centre for Research in Epidemiology and Statistics (CRESS), Université de Paris, INSERM, INRAE, F-75004 Paris, France; mdeago@gmail.com (M.D.A.); barbara.heude@inserm.fr (B.H.)

**Keywords:** handedness, prenatal factors, family handedness

## Abstract

The goal of the study was to investigate some of the factors suspected to be related to children’s handedness: presentation during the last weeks of gestation and at birth (cephalic or breech), side of presentation (right or left), number of weeks of gestation, season of birth, parents’ handedness and sex. We analyzed the relationships between these factors and the child’s handedness at five years. Children (*n* = 1897) from the EDEN cohort participated in the study, among which 1129 were tested for handedness at five. The father’s handedness, but not the mother’s, was significantly related to the child’s hand preference. The percentage of left-handed children was significantly larger when the father was non-right-handed compared to right-handed, and tended to be larger among children in non-left-cephalic presentation compared to left-cephalic presentation. Girls, but not boys, were significantly less lateralized when they were born before 37 weeks of pregnancy than after. Finally, children born in winter or spring were slightly but significantly less lateralized than children born in summer or autumn. All six children who were not lateralized at 5 presented one or several of these factors. These results are discussed in light of the mixed model of handedness.

## 1. Early Appearance of Hand Preference

Whereas right-handedness has been predominant over left-handedness from all time in all societies, the origin of hand preference is still subject to debate. The early manifestation of hand preference argues for a genetic basis of handedness. But at the same time several biological, environmental and cultural factors were shown to affect the direction and degree of handedness, so that a mixed model of genetic and non-genetic influences on handedness is now often proposed [1,2,3]. Many studies focused on the relationships between handedness and perinatal factors such as birth stress, hormonal level of the pregnant mother, presentation of the fetus, season of birth, prematurity, breastfeeding, etc., often considering only one or two factors independently of the others. The goal of this study was to evaluate to what extent some perinatal factors, considered simultaneously, relate to handedness measured at five years of age. The factors considered here are presentation in utero and at birth, side position in utero, season of birth and number of weeks of gestation controlled for birthweight. We also included parents’ handedness and sex of the child, both factors known to be related to hand preference.

First signs of preference for one hand, most often the right one, appears very early in utero, at 15 weeks of gestation in thumb sucking [4,5] and arm movements [6,7], see however [8,9] for different results. The hand preferred by the fetus for sucking appears to be predictive of hand preference of the child for writing at 12 years of age [10]. When infants start grasping objects, a predominance of right-handedness can be observed at the population level [2,11,12,13,14,15,16,17,18]; for a review, see [19]. However, at six months and for the few following months, hand preference during grasping tends to fluctuate at the individual level [20,21,22], at least for the majority of infants [12]. This led one of us to propose that handedness has probably a genetic origin, more or less direct, but that this genetic factor induces only a slight preference toward the right (for most infants) and is reinforced before and around birth to become the powerful characteristic observed in most children and adults [2].

## 2. Presentation/Side

Presentation of the fetus during the last weeks of pregnancy and at birth is one of the environmental factors which could reinforce an early tendency toward right-handedness. More than 95% of fetuses place their head in the mother’s pelvis some time during the third semester (cephalic) [23], whereas around 3% of infants stay in breech presentation until birth, and 0.3% lie horizontally in the uterus (transverse). According to one study including 1102 participants, the majority of cephalic fetuses lie on the left side of the mother (53.2%) whereas 34.8% lie on the right side and 12% are aligned with the mother’s transversal axis [24]. This majority of left-cephalic is probably due to the mother’s anatomical asymmetries [25].

There are reasons to think that left-cephalic presentation could reinforce the use of the right hand, as opposed to right-cephalic or other presentations. When it is in left-cephalic presentation, the fetus has its right hand near the soft tissues of the mother and it might be easier to move the right hand than the left hand. If, in addition, the fetus turns its head to the right more often than to the left, as it has been observed to happen increasingly as gestation progresses [26], then the right thumb is closer to the mouth than the left thumb, and more likely to end up in the mouth for thumb sucking. The tendency to turn the head to the right might even be reinforced by the fact that when lying to the left side and head down, turning the head to the right results in seeing more light than turning the head to the left. It is believed that there is enough light in utero for the fetus to see its arm move [27,28] (see [26] for the difference in head turning to the right between left-cephalic and right-cephalic fetuses). If the right arm in motion is more likely to be seen by the fetus that the left arm in motion, and if the right hand touches the mouth more often than the left hand, this results in the left-cephalic fetus having a combination of proprioceptive, tactile and visual feedback greater for the right hand-arm system than for the left one. This could give an advance to the right hand over the left hand for sensori-motor coordination, since, according to other fetal studies [9,29], the fetus seems able to detect the effect of its own actions on its body from the age of 20–22 weeks.

Interestingly, one study found a significant relationship between side presentation of cephalic children at birth and hand preference at two years of age [30]. In their cohort of 1102 children, the authors observed more left-occipital anterior (LOA, one of the three types of left-cephalic fetal presentations) than right-occipital anterior (ROA) (LOA = 54.4%; ROA = 45.4%). At two years of age, the percentages of right-handed, left-handed and non-lateralized children were 75.9%, 8.4% and 15.7%, respectively. Among the left-handed children, 62.4% were ROA-born, whereas, among the right-handers, only 42.7% were ROA-born. Among the non-lateralized children, the percentage of LOA- and ROA-born was approximately equal. However, another study in which 357 infants with cephalic deliveries were compared for handedness at six years of age, non-right handedness was no more common in ROA- than in LOA-born children [31]. Still another study also found no relationship between fetal presentation and handedness at 7 years, but this study only included three left-handers [32]. Thus, the influence of late fetal position and presentation at birth is still unclear.

## 3. Season of Birth

A few studies have shown an effect of season of birth on handedness. In a meta-analysis including a total of 39,379 participants [33], the authors found a significant tendency for the incidence of left-handers to be higher among persons born in March to July in the Northern hemisphere, in men but not in women. The anisotropy was reversed in the Southern hemisphere. However, this meta-analysis was criticized, in particular for combining studies using very different methodologies [34]. In fact, other large studies did not found a significant difference in the relative frequency of left- and right-handers depending on when they were born during the year [35,36], or they found a different effect. For instance, in one study it was found that boys born during the autumn and winter months (September–February) were more often non-right-handed than boys born in the spring or summer months [37]. Similarly, a recent study including 7658 participants, with a replication study of 5062 participants [38], observed a surplus of left-handed men born in the period November–January compared to the rest of the year. But in a study involving a UK biobank with ≈500,000 participants, an effect of season of birth was observed in women but not in men, and this time left-handedness was associated with being born in the summer [39,40]. What appears from these studies is that the effect of the season, if any, is weak enough so that only very large cohorts allow it to be observed. It is possible anyhow that a small percentage of the small percentage of left-handedness or ambidexterity would be influenced by the season.

Season of birth could influence handedness through the fetal experience during the last weeks of pregnancy for at least two reasons. First, hormone level varies with the length of photoperiod, and hormone level has sometimes been shown to influence brain lateralization for language and handedness [41], with sex differences regarding this effect [38,42]. Second, a combination of more sun and lighter clothing of the mother during the warm seasons might induce more reinforcement of a right-hand tendency due to a better sight of their moving right arm by fetuses born during these seasons. The two explanations are not exclusive of each other. The effect of the season of birth on handedness as a function of sex was included in this study.

## 4. Number of Weeks of Gestation

Several studies on infants and children show that handedness is less well established in preterm infants that in full-terms [43,44,45,46,47]. Despite the fact that not all studies reported significant differences between preterm and term infants, most of them indicate less right-handedness in the former than in the later. Domellöf, Johansson, and Rönnqvist [48] have reviewed 21 studies published between 1980 and 2010, involving a total of 9560 children tested for handedness at age 3 to 19 (773 male and 825 female preterm children; 4138 male and 3824 female control children) and including a meta-analysis from 18 of these studies (1947 preterm infants, 9170 control infants). They observed that there is an approximate two-fold increase in left and/or non-right handedness in preterm children compared with full-term controls.

It is not clear whether it is the low gestational age per se or the low birthweight, often associated with prematurity, that correlates the most with non-right handedness. A few studies indicate that very low birthweight is more important than gestational age in favoring non-right handedness. Two studies found that preterm infants with extremely low birthweight (ELBW) deviate particularly from typical right-handedness [49,50]. In another study on 2252 triplets tested at age 3 to 15 [51], a higher frequency of left-handers was observed in triplets than in singletons even after controlling for gestational age, and there was an inverse continuous relationship between the frequency of left-handedness and birthweight, thanks to many triplets weighting less than 1.5 kg at birth [39]. In addition, one study observed that the difference between singletons and twins –more left-handers in twins- disappears after controlling for birth-related factors, including birthweight (and Apgar score and gestational age) [52]. In fact, in Heikkila’s research, birthweight was significantly associated with an increase of left-handers, not linearly but when contrasting lower birthweight (<2060 g for boys and <2000 g for girls) and higher birthweight (>2060 g for boys and >2000 g for girls). The former included more left-handers than the later, significantly in preterm infants.

Number of weeks of gestation could influence handedness in that children born before term could lack some of the reinforcement of the right hand-arm system of the last weeks of gestation. If this reinforcement is due to the fact that, by the last few weeks, the fetuses are stuck in a position where they have more reinforcement of their right arm, then having a very low weight, independently of the age of gestation, may give more possibility to turn around and to have less asymmetrical experience.

Thus, we investigated: (1) the distribution of cephalic versus breech and transverse presentations during the last weeks of gestation and at birth, of left and right side position of the cephalic fetuses seen at the third trimester’s ultrasound recording, of number of weeks of gestation controlled by birthweight, of season of birth, and (2) whether these factors were related to the direction and the degree of handedness of the child tested at five years of age. (The direction of handedness refers to the side of the preferred hand (right or left), whereas its degree refers to amplitude of the preference for the preferred hand.) To these four factors, we added parents’ handedness and sex, both having been related to handedness [53,54,55,56] (for family handedness); [57,58,59], (for sex effect).

## 5. Methods

### 5.1. Participants

Longitudinal data came from the French EDEN cohort [60]. The EDEN study is a birth-cohort study that aims to investigate the role of pre- and post-natal determinants on child growth, development, and health. Between 2003 and 2006, pregnant women were recruited before 24 weeks of amenorrhea, in 2 French university hospitals in Nancy and Poitiers. Exclusion criteria were multiple pregnancies, illiteracy, and plans to move outside the region in the next 3 years. In addition, women whose health condition might impact the brain development of their fetus were eliminated: this included known diabetes prior to pregnancy, heart conditions, addictions, hormonal disorders). Among women who fulfilled these inclusion criteria, 55% agreed to participate (*n* = 2001). The study was approved by the ethical research committee (Comité Consultatif de Protection des Personnes dans la recherche biomédicale) of the Hospital Bicêtre, and by the Data Protection Authority (Commission Nationale de l’Informatique et des Libertés). Informed written consents were obtained from the parents at enrollment for themselves and for the newborn after delivery.

### 5.2. Data Collection

We report here the distribution of each factor in the cohort (cephalic or breech presentation at the third trimester and at birth, side of fetal position at the third trimester, in particular for cephalic children, number of weeks of gestation and birthweight, season of birth), in addition to parents’ handedness and sex. We also report the children’s handedness at five years of age.

Children’s handedness was evaluated at 3 and 5 years of age using an 8-item Hand Preference Test (writing, erasing, cutting with scissors, holding the spoon to eat, cutting with a knife, holding a hair brush, holding a comb, holding a hammer) [61]. The possible responses were right hand, left hand or either one. Children’s data for handedness were collected by trained psychologists in the two study centers (Nancy and Poitiers). In order to investigate which factors are related to hand preference we used handedness at 5 years only, when it is more biased toward the right and closer to the adult’s pattern. Parent’s handedness was evaluated using a questionnaire at the beginning of the study [62].

At birth, there were 1897 children (901 boys, 47.5% and 996 girls, 52.5%). Handedness of the mother is known for 1851 children, father’s handedness is known for 1616 children and both parents’ handedness is known for 1575 children. We have data for side position (left vs. right) at the third trimester of pregnancy for 1656 children, for side position at an additional ultrasound recording for 444 children, for presentation at the third trimester (cephalic vs. breech vs. transverse) for 1819 children, for presentation at birth for 1893 children, for number of weeks of gestation and season of birth for 1897 children. For 1129 children (603 boys and 526 girls) we have data for handedness at 5 years (see Table 1).

## 6. Analysis

Since we did not have data for all 1897 children for all variables, we first analyzed each variable independently on the maximum number of children. We did that to have the best possible representation of the frequency of each variable in this large population of children. Then, we investigated which variables (also referred to as “factor”) were related to handedness at 5 years, indicating the number of children concerned for each analysis.

For handedness, for the children as for their parents, a handedness index (HI) was calculated as the following: [(Number of RH (responses) − Number of LH)/(Number of RH + Number of LH + Number of Either hand)]. We used this continuous index whenever possible. Otherwise we categorized everyone according to the following criteria: children or parents with HI ≥ 0.30 were considered as right-handed whereas those with HI ≤ –0.30 were considered as left-handed. Everyone with HI falling between these two values were considered as non-lateralized or ambidextrous [45,63]. We refer to this category as “non-lateralized” when children are considered, since the process of handedness may not be mature yet, and to “ambidextrous” for parents. For some analyses, we contrasted right-handers (HI ≥ 0.30) with non-right-handers (HI < 0.30). The absolute handedness index (absHI) was also calculated as the following: [(abs (Number of RH − Number of LH))/(Number of RH + Number of LH + Number of Either hand)].

To check the significance of uneven distributions of prenatal variables, we used binomial tests. For handedness at five, we checked the distribution of children using geometric data analysis (GDA; [64]). The basic data set is an Individuals × Responses-to-item table, where each item constitutes a variable composed of three categories, i.e., “right”, “left”, “either one.”

To evaluate to what extent the different independent variables, or factors (presentation, side, season of birth, etc.) are related to handedness at 5 years of age, we made a multivariate analysis for HI and a univariate analysis for absHI. We did a general model non-linear analysis on HI (Probit model), with the category of handedness of the child at 5 as dependent variable and prenatal factors and parent’s handedness as independent variables. In addition, we crossed the prenatal variables with children’s handedness of the basic data set, with the GDA method of multiple correspondence analysis (MCA), more precisely a variant of MCA called specific MCA.

We could not do the same general model non-linear analysis with absHI because this analysis requires that the dependent variable be categorical (plus at least one linear independent variable). This is not a problem with HI which can be meaningfully categorized (right-handers if HI > 0.30 and non-right-handers if HI < 0.30), but for absHI, the categorization into lateralized and non-lateralized results in too few children being non-lateralized to be useful: Thus we made univariate analyses on absHI, using U of Mann-Witney or Kruskal-Wallis tests and Spearman correlations. We also calculated the effect size using Cohen’s *d* [65]. We reported the values superior to 0.20, which is the limit for a small size effect. In addition, we looked at how many of these factors were present in the non-lateralized children.

## 7. Results

### 7.1. Frequency of the Prenatal Variables Studied Here

#### Presentation at the Third Trimester and at Birth (Cephalic vs. Breech vs. Transverse)

Presentation of the fetus was observed at the third trimester at some point between 28 and 38 weeks (*n* = 1819), and at birth (*n* = 1893). At the third trimester most fetuses were in cephalic presentation (*n* = 1605; 88.2%), some were in breech presentation (*n* = 179; 9.8%) and 35 (1.9%) lied transverse. A binomial test indicated that the 88.3% probability of being in cephalic presentation with a 95% confidence interval [0.885; 0.914] was significantly above chance, *p* = 0.0000045. At birth, out of the 1893 infants, the percentage of cephalic infants was even larger (*n* = 1817, 96%) and the percentage of breech infants was lower (*n* = 71; 3.8%). For five children, data about birth presentation were missing, 4 of them being born after a caesarian section. A binomial test indicated that the 96% probability of being in a cephalic presentation at birth with a 95% confidence interval [0.952; 0.970] is significantly above chance, *p* = 0.000000. Out of the infants for which we know the presentation at the third trimester and at birth, 1646 were in the same presentation at both observations: 123 fetuses were in breech or transverse presentation at the third trimester of pregnancy and cephalic at birth. Less infants changed from cephalic to breech or transverse presentation during the last weeks of pregnancy (*n* = 14). Finally, of the 35 transverse fetuses at the third trimester, 34 were in cephalic presentation at birth and one was in breech presentation. Thus, some children turned head-down quite late during pregnancy. In fact, the presentation of the fetus at the third trimester was related to age at the time of the observation: the mean age of the breech fetuses (31 weeks, SD = 1.28) was significantly lower than the mean age of the cephalic fetuses (32 weeks, SD = 1.1), F (1, 1784) = 17.6, *p* = 0.00002.

Thus, cephalic presentation was the most frequent, both at the third trimester and at birth, but when children were in breech or transverse presentation at the third trimester they could still change for a cephalic presentation before birth (the reverse was rarer). We decided to analyze handedness as a function of presentation at birth only: cephalic children at birth were opposed to breech at birth. The children who were born in cephalic presentation but had been in breech or transverse presentation at the third trimester’s ultrasound recording were excluded from the handedness analysis, as well as the children who were born in breech presentation after being cephalic at the third trimester. 1646 children were thus considered, out of which 1590 were in cephalic presentation (96,5%) and 56 were not.

### 7.2. Side Position at the Third Trimester

Out of 1656 fetuses, 948 (57.2%) lied with their back to the left side of the mother. When an additional ultrasound recording was made (*n* = 444), a majority of children were lying to the left of the mother (*n* = 239; 53.8%). A binomial test indicated that the 53.8% probability of lying to the left of the mother with a 95% confidence interval [0.522; 0.616] was significantly above chance, *p* < 0.05. However, out of the 369 children for whom we had data both at the ultrasound recording of the third trimester and at an additional ultrasound recording, only 139 were lying to the left of the mother at both recordings (37.7%), 72 (19.5%) were lying to the right of the mother at both recordings, 79 children changed position from right to left (21.4%) and 79 children changed position from left to right. The group of children lying to the left side of the mother at both recordings remained higher than the three other groups. A binomial test indicated that, compared to the theoretical value of 25%, the 37.7% probability of lying to the left of the mother at both assessments with a 95% confidence interval [0.330; 0.426] was significantly above chance, *p* < 0.05. In contrast, a binomial test indicated that the 19.5% probability of lying to the right of the mother at both assessments was not different from chance, *p* = 0.45. Thus, even though lying with its back to the left side of the mother at the third trimester could still change before birth, more children lied to the left side at both assessments than at the right side at both assessments.

Considering only the cephalic children, a majority of the 1475 fetuses for whom side position at the third trimester was known lied with their back to the left side of the mother at the third trimester (*n* = 852, 57.8%). 623 cephalic fetuses (42.2%) lied with their back to the right side of the mother. A binomial test indicated that the 57.8% probability of lying to the left of the mother with a 95% confidence interval [0.397; 448] is significantly above chance, *p* < 0.05.

Out of the 1511 children for whom we had data for side of lying at the third trimester and presentation at the third trimester and at birth (excluding children whose presentation differed at both assessments), 843 were lying to the left of the mother’s womb and were in cephalic presentation (55.8%), 617 were lying to the right of the mother’s womb and were in cephalic presentation (40.8%), and 51 were in breech presentation (25 to the left, 1.65% and 26 to the right, 1.7%).

### 7.3. Number of Weeks of Gestation

The mean number of weeks of gestation at birth of the 1897 children for whom we have data is 39.2 (SD = 1.7, min = 27, max = 42). 112 children had a mean gestational age at birth lower than 37 weeks (5.9%), among them 22 children had a mean age at birth lower than 32 weeks (1.2%). 37 weeks and 32 weeks are the limits fixed by the World Health Organization for moderate and very preterm birth, respectively.

### 7.4. Season of Birth

28.5% of the 1897 children were born in spring, 26.3% in summer, 22.8% in autumn, and 22.4% in winter. A binomial test indicated that the 28.47% probability of being born in spring with a 95% confidence interval [0.264; 0.305] was significantly above chance, *p* = 0.005. The probabilities of being born in autumn or in winter were significantly below chance (autumn: confidence interval [0.209; 0.247], *p* = 0.02; winter: confidence interval [0.206; 0.248], *p* = 0.009). The probability of being born in summer was at chance level.

### 7.5. Parents’ Handedness

Out of the 1851 mothers for whom we have handedness data, 89.3% were right-handed, 9.2% were left-handed, and 1.5% were ambidextrous. Out of the 1616 fathers for whom we had handedness data, 87.7% were right-handed, 9.6% were left-handed, and 2.7% were ambidextrous. The difference between the mean HI of the mothers (HI = 0.77; SD = 0.54) and the mean HI of the fathers (HI = 0.73; SD = 0.54) is not significant. We have andedness data of both parents for 1575 children: 77.6% had two right-handed parents, 11.1% had a right-handed mother and a non-right-handed father (left-handers and ambidextrous), 9.9% had a right-handed father and a non-right-handed mother, and 1.3% had two non-right-handed parents.

## 8. Children’s Handedness at 5

**Figure 1:**
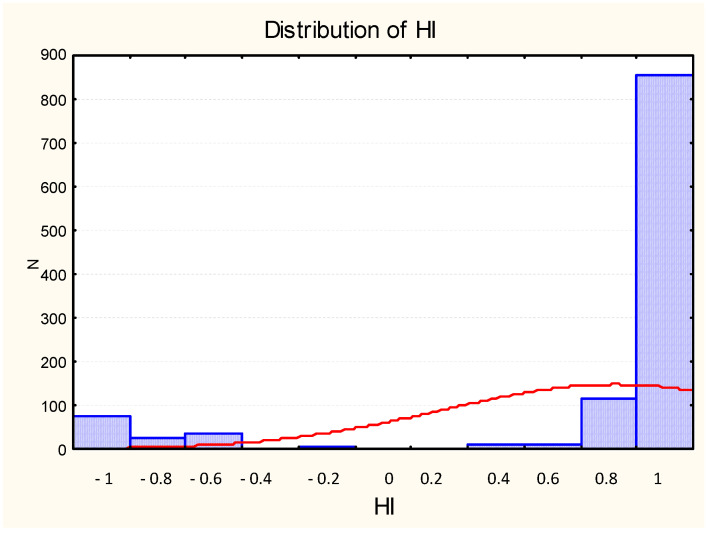
Distribution of HI at 5 years.

The mean handedness index (HI) was 0.72 (SD = 0.61; *n* = 1129) at age 5 (see Figure 1, for HI’s distribution). When the whole population was categorized as a function of their HI, 988 children were right-handed (87.5%), 135 were left-handed (11.9%), and 6 were non-lateralized (0.5%). The mean absHI was 0.93 (SD = 0.13). AbsHI was significantly lower for left-handers (absHI = 0.89, SD = 0.14) than for right-handers (absHI = 0.94, SD = 0.11), z = 3.2, *p* = 0.00008, Cohen’s d = 0.397.

Girls’ HI was 0.73 (SD = 0.59) whereas boys’ HI was 0.71 (SD = 0.61). Girls’ absHI was 0.93 and boys ‘HI was 0.92. There was no significant difference for sex, neither for HI nor for absHI.

**Figure 2:**
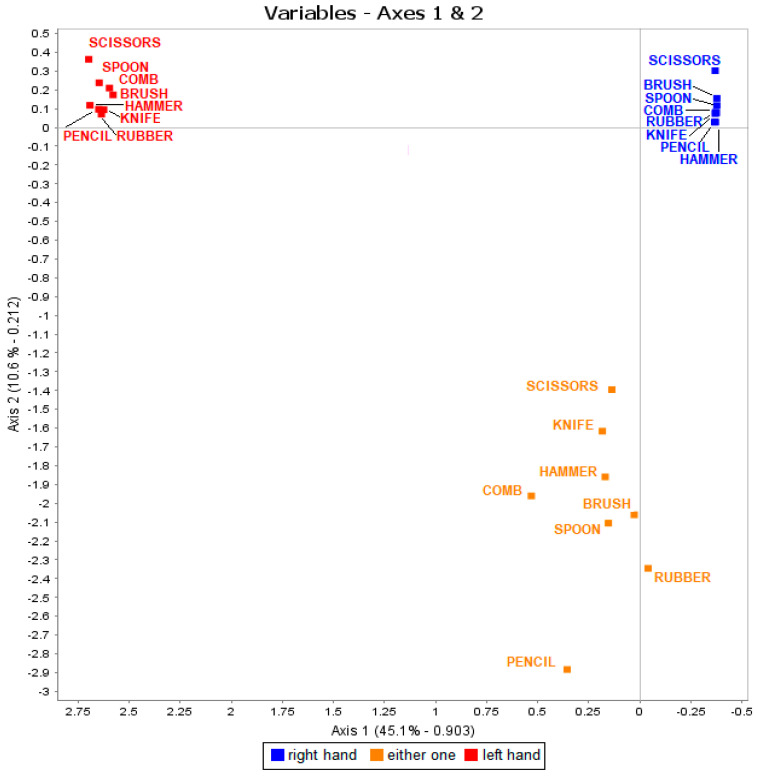
Distribution of Responses to the 8-item Hand Preference Test as a function of hand at 5.

The basic data set of Individuals × Responses-to-item is represented on Figure 2. Axe 1 is the laterality axis, and axis 2 is strength of laterality axis. We see that pencil, rubber and spoon were the three most strongly lateralized items. The first two dimensions (axes 1 and 2) represent 56% of the total variance and 93% importance.

## 9. Multivariate Analysis on the Direction of Handedness (HI) at 5 Years of Age

For the following analyses, only the children for whom we had data for handedness at 5 years of age could be considered. A preliminary analysis showed that sex was never significantly related to HI, neither as a main effect nor as an interacting factor, and that HI did not differ with seasons. Therefore, Sex and Season of birth were not included in the multifactorial analysis of HI.

A general model non-linear analysis was calculated with the child’s category from HI as dependent variable (R-handed vs. NR-handed), and Presentation (Cephalic vs. Breech), Side of position (Left vs. Right), Mother’s handedness (R-handed vs. NR-handed), and Father’s handedness (R-handed vs. NR-handed) as independent categorical variables. There was one independent continuous variable, Number of weeks of gestation. The results showed a close to significant effect for only one factor, Father’s category of handedness, *p* = 0.060. Since there was absolutely no effect for Mother’s category of handedness (*p* = 0.85), we did again the same analysis without this factor. The results showed a significant effect for Father’s handedness, *p* = 0.038, and a close to significant interaction between Presentation, Side of position, and Father’s handedness, *p* = 0.083. Number of weeks of gestation was not significant and none of the other interactions were significant.

**Figure 3:**
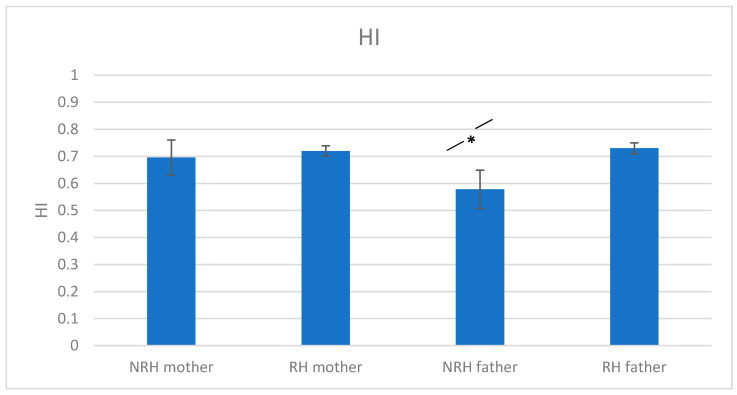
Children’s HI at 5 years as a function of their mother’s and father’s handedness (Error bars represent the SE). * = significant difference (*p* < 0.05).

Thus, the percentage of non-right-handed children varied significantly depending on whether the father was non-right-handed or right-handed: children’s HI at 5 years was higher when the father was right-handed (HI = 0.76; SD = 0.64; *n* = 873) than when he was left-handed (HI = 0.56; SD = 0.80; *n* = 105) or ambidextrous (HI = 60; SD = 0.74; *n* = 23) (see Figure 3). Cohen’s d = 0.28 for the difference between HI of non-right-handed children having a right-handed versus left-handed father, 0.23 for the difference between HI of non-right-handed children having a right-handed versus an ambidextrous father.

**Figure 4:**
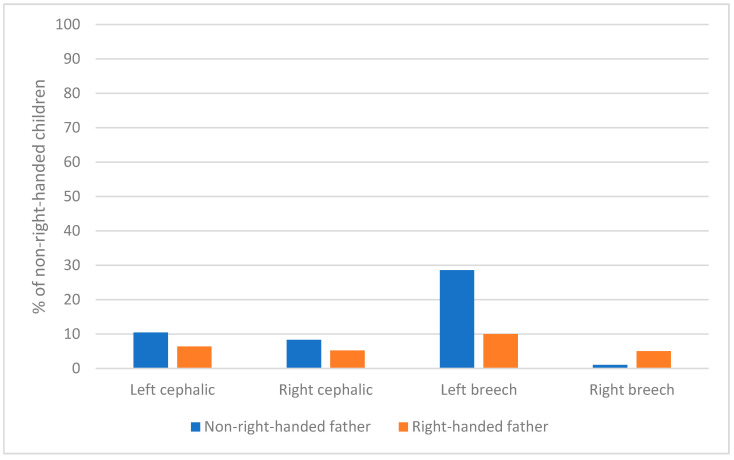
Percentage of non-right-handers at 5 as a function of father’s handedness, presentation and side of position.

In addition, this effect was larger when the children had been in breech presentation and lying to the left than in the other conditions of presentation and side of lying (see Figure 4).

## 10. Univariate Analysis on the Degree of Handedness (absHI) at 5 Years of Age

For sake of clarity, before presenting the results of the univariate analysis on absHI, we mention the value of HI.

### 10.1. Presentation at Birth

Among the 5-year-old children for whom we have both handedness and birth presentation data (*n* = 975, keeping only those children whose presentation was the same at the third trimester and at birth, as already mentioned), 939 were in cephalic presentation (96.7%) and 36 were in breech presentation (3.7%). As we saw, presentation was not significantly related to the child’s category of handedness as a main factor, even though the HI of the children was higher when children had been in cephalic presentation (0.72, SD = 0.60) than in breech presentation (0.59, SD = 0.72). There was almost no difference in absHI between cephalic and non-cephalic (cephalic: absHI = 0.93, SD = 0.13; breech: absHI = 0.91, SD = 0.14), so that we did not test it statistically.

### 10.2. Side of Position and Presentation at Birth (Left Cephalic versus Non-Left Cephalic)

As already mentioned in the introduction, lying to the left results in the fetus having the right arm toward the soft tissues and more light than the right side, but this is true for children in a cephalic presentation only. As we saw in the previous section, side of position was not significantly related to the child’s category of handedness as a main factor, but had an effect in interaction with presentation at birth and father’s handedness. Thus, for this analysis, we contrasted left-cephalic children with non-left-cephalic children.

Among the 5-year-old children for whom we have data on handedness, presentation and side of position at the third trimester (*n* = 895), 511 were left-cephalic (57.1%, HI = 0.74); 350 were right-cephalic (39.1%, HI = 0.71), 19 were left-breech (2.1%, HI = 0.51), and 15 were right-breech (1.7%, HI = 0.98). There was a slight tendency for the left-cephalic children to have a higher HI (0.74; SD = 0.58) than non-left-cephalic children (either right-cephalic or breech, HI = 0.70; SD = 0.64). AbsHI was similar for left-cephalic fetuses and non-left-cephalic fetuses (0.93) so that we did not test it statistically.

### 10.3. Number of Weeks of Gestation

As opposed to the absence of effect of the number of weeks of gestation on HI, as we saw previously, absHI correlated slightly positively, albeit significantly, with the number of weeks of gestation, r = 0.10, *p* < 0.05. The higher the number of weeks *in utero*, the higher the absHI. The effect was due to girls: the absHI of girls was higher when they were born after 37 weeks or more (0.94, SD = 0.12), than when they were born after 32 to 37 weeks (0.87, SD = 0.19), or after less than 32 weeks (0.79, SD = 0.3). The Mann-Whitney test showed that the difference was significant between girls born after 37 weeks or more and girls born after less than 37 weeks, z = 2.39, *p* = 0.017. Cohen’s d = 0.44 for the difference between absHI of girls being born after 37 weeks or more and of girls being born after 32 to 37 weeks; 1.71 for the difference between absHI of girls being born after 37 weeks or more and of girls being born after less than 32 weeks; 0.59 for the difference between absHI of girls being born after 32 to 37 weeks, and of girls being born after less than 32 weeks.

To check the possible confounding effect of birthweight, we made a regression analysis including not only prematurity but also birthweight, with absHI as dependent variable. The result showed a significant global effect, *p* = 0.0000, a significant effect for Prematurity, *p* = 0.006, beta = −0.08, but no significant effect for Birthweight, *p* = 0.15, beta = −0.04. Thus, duration of pregnancy was related to girls’ degree of handedness, an early birth being significantly associated with less manual lateralization at five years. Birthweight was not a confounding effect.

### 10.4. Season of Birth

As we already mentioned, HI did not differ significantly with season of birth. AbsHI was slightly lower for the children born in winter or in spring than for the children born in summer or in autumn. A Mann-Whitney test showed that the difference was significant, z = 4.23, *p* = 0.0002. (see Table 2). Cohen’s d = 0.39 for the difference between absHI of children being born in winter and of children being born in autumn; 0.22 for the difference between absHI of children being born in winter and of children being born in summer; 0.34 for the difference between absHI of children being born in spring and of children being born in autumn. All other values of Cohen’s d are below 0.20, and thus negligible.

Thus, season of birth was, to a very small extent, related to the degree of handedness.

### 10.5. Parents’ Handedness

As already mentioned, children’s direction of handedness at 5 years did not differ whether the mother was right-handed or non-right-handed, but it was related to the father’s handedness. AbsHI was not different depending on parent’s handedness, mother’s and father’s alike, so that we did not test it statistically.

## 11. Ratio of Right-Handed to Left-Handed Children at Age 5 as a Function of All Factors

In our multivariate analysis on the direction of handedness, we considered the effect of prenatal factors on being right-handed or not-right-handed, including children who were not lateralized. But one question behind this study was “how can one explain that most people are right-handed, and that around 10% become left-handed”. Thus, in the next two sections, we shall consider separately left-handed and ambidextrous children.

Given the results so far, we cannot expect prenatal factors to account much for the left-handedness of the child at five: only the father’s handedness was significantly related to the children’s HI, but even the children of non-right-handed fathers had, on average, a HI well above 0, and therefore, as a group, used more their right than their left hand. They were merely less right-handed than the children whose father was right-handed. However, since the variance was more important among children of a non-right-handed father than among children of a right-handed father, it is not impossible that a greater percentage of left-handed children had a left-handed father, compared to the percentage of right-handed children having a left-handed father. In addition, we looked at the combined effect of having a non-right-handed father and non-being in left-cephalic presentation since left-cephalic children tended to have a higher HI than non-left-cephalic children.

### 11.1. Father’s Handedness

**Figure 5:**
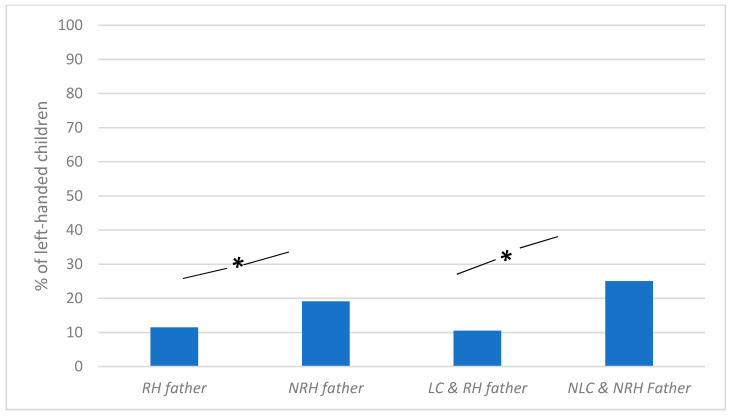
Percentage of left-handed children as a function of father’s handedness (RH = right-handed; NRH = non-right-handed) and father’s handedness and side of position combined (LC = left cephalic; NLC = non-left-cephalic). * = significant difference (*p* < 0.05).

The percentage of left-handers was significantly smaller among children of a right-handed father (11.5%) than among children of a non-right-handed father (19.1%), X^2^(1) = 5.8, *p* = 0.016) (see Figure 5). Said differently, 16.7% of left-handed children had a non-right-handed father against 9.8% of right-handed children.

### 11.2. Presentation and Side Position, and Father’s Handedness Combined

The percentage of left-handers was significantly smaller among left-cephalic children of a right-handed father (10.5%) than among non-left-cephalic children of a non-right-handed father (25%), X^2^(1) = 6.7, *p* = 0.009) (see Figure 5).

**Figure 6:**
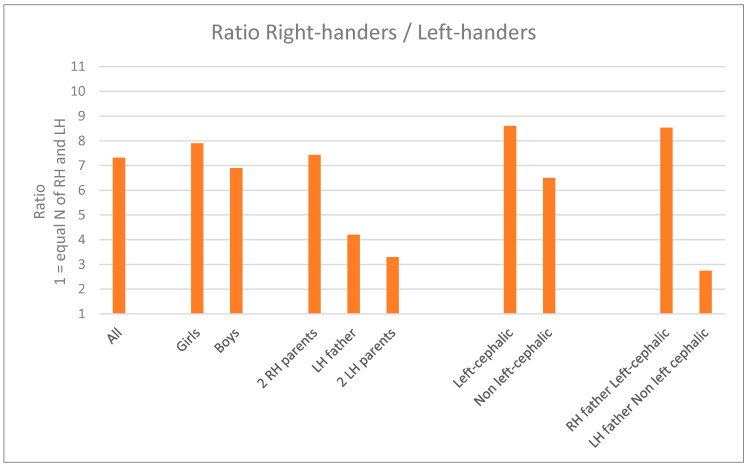
Ratio between the number of right-handers and of left-handers at 5 as a function of the main factors studied (*n* = 1123 for all data and for sex; 761, 104 and 13 for parents’ handedness, 508 and 382 for Left-cephalic and Non left-cephalic, and 391 and 30 for RH father Left-cephalic and LH father Non left cephalic). The lowest the ratio, the more equal the number of right-handers and left-handers.

Finally, Figure 6 shows the ratio between right-handers and left-handers children as a function of the main factors related to handedness at five, to which we added sex.

**Figure 7:**
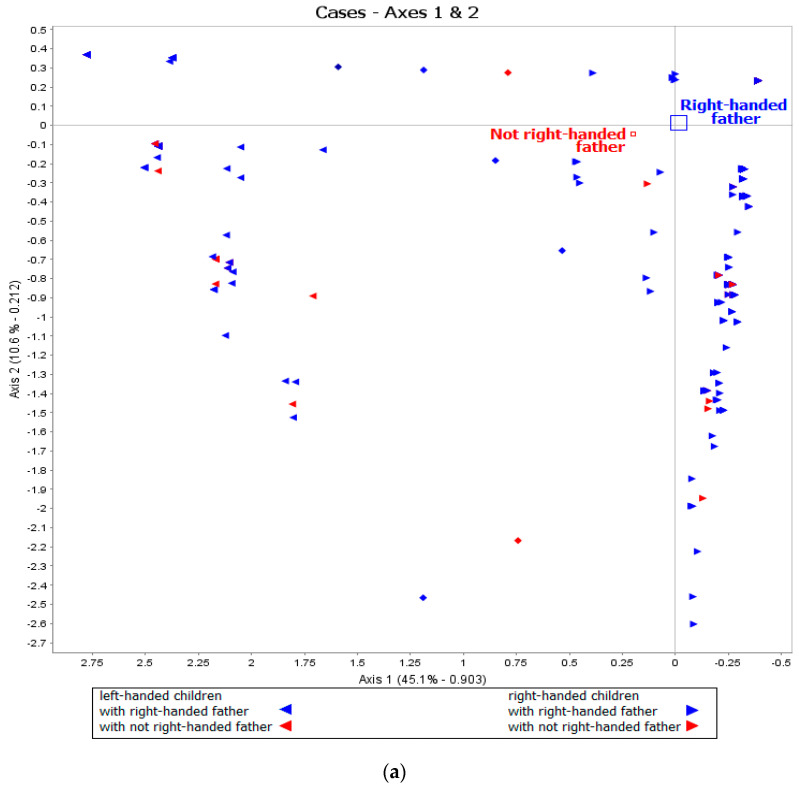

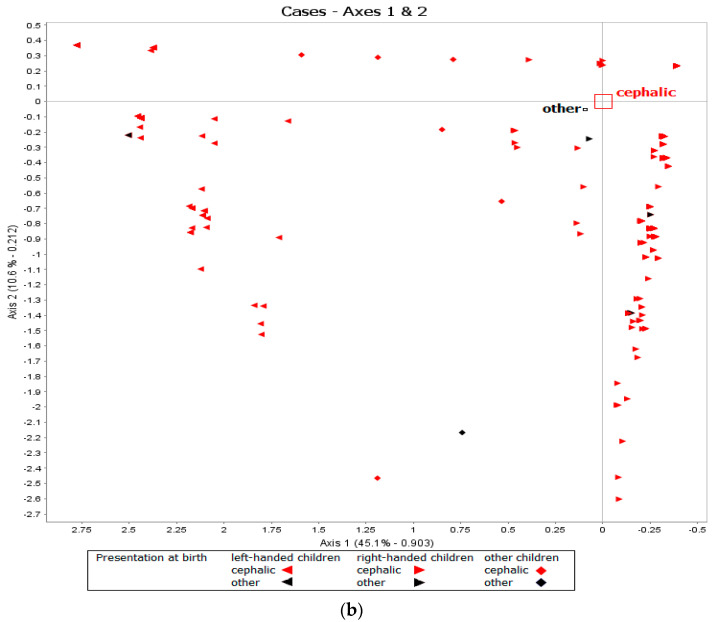
(**a**) Distribution of left-handed and right-handed children (X axis) as a function of father’s handedness (Y axis). (**b**) Distribution of left-handed and right-handed children (X axis) as a function of presentation at birth (Y axis).

We also used the GDA method of multiple correspondence analysis to represent how the three groups of right-handed, left-handed and non-lateralized children at 5 differed with respect to the father’s handedness and presentation and side of presentation at birth (see Figure 7a,b). One sees that neither of the two factors were sur-represented among left-handers compared to right-handers.

**Figure 8:**
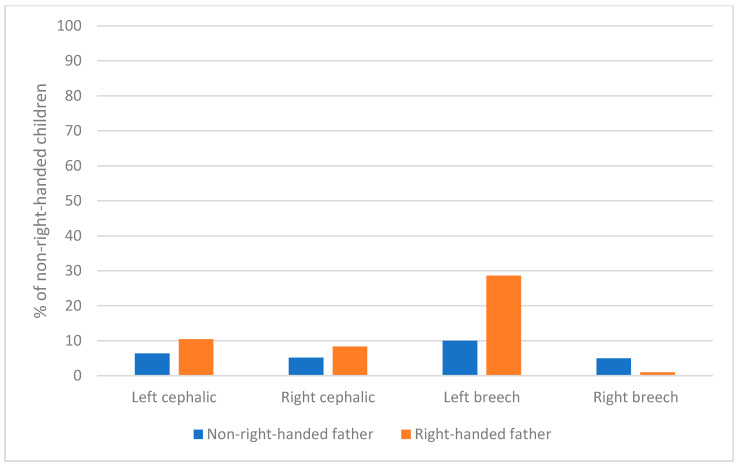
Distribution of the 12% left-handed children at 5 as a function of fathers’ handedness and presentation and side of presentation (*n* = 96).

We finally looked at the distribution of left-handers as a function of the two factors cited above, father’s handedness and presentation and side of presentation. We have data for children’s handedness at 5, as well as for father’s handedness and for side position and presentation in utero and at birth for 796 children, including 696 right-handers (87.4%), 96 left-handers (12.1%) and 4 non-lateralized children. The 96 left-handed children were distributed as follows: 9 had a non-right-handed father and were not in left-cephalic presentation; 9 had a non-right-handed father and were in left-cephalic presentation; 37 had a right-handed father and were not in left-cephalic presentation, and 41 had a right-handed father and were left-cephalic presentation (see Figure 8). Thus, for almost half of them (41/96, 42.7% of them), handedness could not be associated to either of these two factors. 42.7% is not very far from the 50.3% of right-handers that had a right-handed father and were in left-cephalic presentation. Thus, these factors are neither necessary nor sufficient to explain left-handedness.

## 12. Difference between Lateralized and Non-lateralized Children: Qualitative Analysis of the 6 Non-Lateralized Children

Finally, to try to understand why some children were still non-lateralized at 5, we made a qualitative analysis of the results of the six non-lateralized children (see Table 3). The six non-lateralized children were either born in winter or spring (*n* = 5) or born after less than 32 weeks of gestation (*n* = 1), or had a non-right-handed father (*n* = 2) (we added on Table 3 that one child had a left-handed mother), or were in breech presentation at birth (*n* = 1), or, if cephalic, were right-cephalic (*n* = 1). Four children had 2 or more of these factors.

## 13. Discussion

The goal of the study was to investigate the effect of prenatal factors which have been shown to be related to children’s handedness; in contrast to most of the previous studies in which these factors were studied separately, we considered these factors within the same study. The factors included: (1) presentation at birth (cephalic versus breech and transverse) if not different from presentation at the third trimester, (2) side of presentation at the third trimester for the cephalic fetuses (right versus left), (3) number of weeks of gestation controlled by birthweight and (4) season of birth. To these four factors, we added sex and parents’ handedness, both having been shown to be related to handedness.

Prenatal and postnatal data came from the French EDEN cohort. We report here the results from children observed at the ultrasound recording of the third trimester (sometimes twice), at birth for 1897, and at age 5 for 1129 of them for handedness assessment. We first analyzed each factor individually, to evaluate their frequency in our population, and then we related these factors to the child’s handedness at 5.

Regarding the presentation of the fetus, cephalic presentation was the most frequent, both at the third trimester and at birth. When children were in breech presentation (or transverse) at the third trimester they could still change for a cephalic presentation before birth. A change from cephalic to breech was very rare. The frequency of 96% of cephalic children and 3.8% of breech at birth is in accordance with recent data [23].

At the third trimester, significantly more cephalic fetuses lied with their back to the left side of the mother (57.8%) than with their back to the right side of the mother (42.2%). Some fetuses changed side during the third trimester and only 37,7% were seen lying to the left of the mother at both ultrasound recordings when an additional recording was available. However, lying to the left of the mother at both recordings was the most frequent of the four possibilities (LL, RL, LR, RR). Thus, even though lying with its back to the left side of the mother at the third trimester could still change several times before birth, significantly more children lied to the left at both assessments than to the right at both assessments. This relatively small but significant majority of cephalic children lying to the left side of the mother fits with the numbers reported elsewhere [24].

5.9% of the children were born after less than 37 weeks of gestation. This number is lower than the 10% indicated by the World Health Organization, but relatively close to the 7.4% given by the French institute of health and medical research (INSERM). Among the preterm children, 4.7% were born after 32 weeks of pregnancy or more, thus were moderately preterm, and 1.2% were born after less than 32 weeks, and thus could be considered as very preterm.

The probability of being born in one of the four seasons was close, with however significantly more children born in spring than the chance percentage of 25%, and significantly less children born in autumn or winter than chance for both seasons. Birth distribution according to season varies with geography and culture but a higher rate of birth in spring and a lower rate in winter has been observed several times in France (e.g., [66]).

Regarding parents’ handedness, 10.7% of the children had a non-right-handed mother, 12.3% had a non-right-handed father, and 1.3% had two non-right-handed parents. The lower percentage of non-right-handed mothers, as compared to the percentage of non-right-handed fathers, fits well with the literature showing that women tend to be more right-handed than men, albeit non always significantly (see [59] Papadatou-Pastou et al., 2008, for a review). There were no relationships between the mother’s handedness and the presentation of the child.

Thus, the majority of the children of the EDEN cohort were cephalic with their back to the left side of the mother (rather than right-cephalic or breech), born after 37 weeks of gestation or more, and from two right-handed parents.

Since some studies showed an effect of ultrasound exposure on handedness in males, we checked whether the additional ultrasound at the third trimester of some of the children could have had an impact on the handedness index at five and on the number of non-right-handed children. In our study, there was no significant effect on HI of the additional ultrasound recording, neither in males nor in females. However, as was observed in Kieler et al.’s study [67] or Torloni et al.’s meta-analysis [68], the males whose mother was exposed to an additional ultrasound recording had a HI slightly inferior to those whose mother was not exposed to the additional recording. This was not true for females.

Secondly, we evaluated the handedness of the children at 5 years of age. The mean HI was 0.72, which indicates that, as a mean, the right hand was largely preferred to the left one. With 87.5% of right-handers and 11.9% of left-handers, the 5-year-olds of the EDEN cohort have a distribution according to handedness very close to what is observed in the adult population when there is no pressure against left-handedness (e.g., [63,69]).

Thirdly, we evaluated whether the prenatal factors cited above were related to handedness of the child tested at five years, adding sex as another independent variable. For the direction of handedness, we used HI or the categories derived from HI, and for the degree of handedness we used absHI.

The multivariate analysis made on the direction of handedness (HI) showed that handedness of the father, but not of the mother, was significantly related to the direction of handedness at 5 years: children are more often right-handed if the father is right-handed than if the father in non-right-handed. When presentation at birth and side of fetal position were combined with father’s handedness, the interaction between the three variables and the child’s handedness was close to significant: children in breech presentation lying to the left had the largest difference of HI depending on the father’s handedness.

The univariate analysis on the degree of handedness (absHI) showed significant effects for duration of pregnancy and season of birth. Duration of pregnancy was significantly related to the degree of handedness: birth after less than 37 weeks of pregnancy was associated with a lower absHI at five, compared to birth after more than 37 weeks. The low birthweight which accompanies shorter pregnancies did not account for this reduced lateralization. However, this effect was observed in girls only. Season of birth was significantly related to the degree of handedness: children born in winter or in spring had a slightly lower absHI at five compared to children born in summer or in autumn. The difference was very small but significant.

### How Can One Interpret These Results?

One factor was significantly related to the direction of handedness: father’s handedness. The significant effect of a parent’s handedness on the child’s own hand preference is well known [53,54,55,56] and interpreted in terms of genetic [70,71] or possibly also imitation-related [72] factor. However, here we found that only the father’s non-right handedness, and not the mother’s non-right-handedness, was significantly related to the child’s hand preference at five. In the literature, when a difference between the effect of father’s and the mother’s handedness was found, it was frequently the mother’s handedness which was more important [73,74,75,76]. However, our results fit with other studies showing that, among non-right handed children, there are more left-handed fathers, but no more left-handed mothers, than among right-handed children [44,77], both sets of results concerning extremely preterm children. If more data confirm the specific effect of the father’s handedness on the child’s own hand preference, the question will remain to understand the genetic, or pre or postnatal environmental mechanisms which could explain it.

For presentation at birth and side of cephalic presentation of the fetus, the tendency we observed in this study for the direction of handedness, bearing on more than 800 fetuses, goes in the expected direction according to the few existing studies. To our knowledge, only Smart et al.’s study [31] looked at the influence of birth presentation on handedness a few years later. Our finding that breech presentation was associated with a tendency toward less right-handedness at 5 fits with their results, except that their finding is restricted to boys. Another study [30], observed slightly less non-right-handers in left-cephalic children than in other presentations, which fits with our own observations. As already mentioned, left-cephalic fetuses have their right hand toward the soft tissues of the mother during the last weeks of pregnancy and since the fetal head is more often turned toward the right [26], they also have more light and more opportunity to see their right hand and touch their mouth with their right hand than right cephalic or than breeches fetuses. Visual, tactile, and proprioceptive feedback from their spontaneous arm movements may give an advance to their right-arm coordination skill compared to the left one. We cannot dismiss completely this factor based on the fact that our results do not reach significance. For instance, there is now a consensus that sex is one factor related to handedness, despite the fact that many studies did not find a significant effect of sex. The facts that most studies go in the same direction (a greater percentage of girls being right-handed compared to boys), and that, understandably, meta-analyses found a significant effect for sex, means that a sex effect is weak and needs very large cohort to emerge significantly. It could be the same with side of left-cephalic presentation, but more studies are clearly needed to draw conclusions.

Thus, contrary to our hypothesis, the general model analysis showed little cumulative effects of the factors. One explanation might be that the factors were cumulated for too few children, partly due to missing data: for instance, only six children had two left-handed parents and were born right-cephalic, none of the children had two left-handed parents and were born before 37 weeks, six children had a left-handed father and a right-handed mother and were born in breech presentation, 50 had a left-handed father and a right-handed mother and were born in right-cephalic presentation, etc.

Two factors were significantly related to the degree of handedness (absHI). The first one, duration of pregnancy, is interesting to relate to the many studies showing that preterm children tend to be less lateralized than term children (e.g., [43,44,45,46,47,48]). Even though we did not find a two-fold increase of the percentage of non-right-handers in preterm compared to full-term children, as shown in Domellöf et al.’s meta-analysis [48], the significantly lower absHI in children born after less than 37 weeks as compared to full-term children goes in that direction. This is in line with a recent study showing a large percentage of non-right-handers in 4–8 year olds formerly extremely preterm [77]. If one considers that the last weeks of pregnancy are important for reinforcing a tendency toward right-handedness [2], then one could expect that lacking the last weeks of pregnancy could result in a lower absHI. For instance, an untimely exposure to extra-uterine factors may interrupt the normal process of neural connectivity and the normal process of cross-talk between hemispheres. Resting-state fMRI fetal studies showed a significant increase in functional coupling between subcortical nuclei and cortical networks in several areas, including areas related to sensorimotor processing in the last trimester of pregnancy [78]. Long-range cortical-cortical connections also have been shown to develop during the third trimester of pregnancy [79], and modules corresponding to left-lateralized regions appear to develop during the last trimester [80]. It is known that there is an asynchrony in the maturation of the two hemispheres (e.g., [81]). Some have proposed the hypothesis of a cognitive blueprint through which primary features develop before birth, in particular during the last trimester of pregnancy. This framework could help understand why premature birth is associated with a deficit in sensorimotor asymmetry, why it is also associated with many developmental disorders, and why so many developmental disorders are associated with a deficit in asymmetry. The fact that this effect was observed only in girls is interesting to compare with Marlow et al.’s findings [82]: in their study, the authors observed that extremely preterm children evaluated for hand preference between 2 and 11 years of age had a lower handedness score than their term counterparts, the between-group effects being consistent through development and with larger differences in females than in males.

Finally, the fact that season was related to the degree of handedness, with more lateralized children born in summer and autumn than in winter and spring, cannot be in accordance with all former studies since many of them gave different results: the season effect was found to vary sometimes between the north and south hemisphere, to differ between men and women; the months of March to July have been noticed to favor left-handedness, at least in the north hemisphere in men, fall and winter months to favor non-right handedness in boys, the months of September to January to favor non-right-handedness in men, summer months to favor left-handedness in women, sometimes no effect was found, etc. (see [33], for a meta-analysis). As mentioned in the introduction, season of birth could influence handedness through fetal experience during the last weeks of pregnancy because hormone level varies with the length of photoperiod, and hormone level has sometimes been shown to influence brain lateralization for language and handedness [41]. More generally, it would have been interesting to know the mother’s hormonal level to interpret our results [41]. For the season-related findings, a combination of more sun and lighter clothing of the mother during the warm seasons might also induce more reinforcement of a right-hand tendency due to a better sight of their moving right arm by fetuses whose last weeks in utero occur during these seasons. Finding more lateralized children in summer and autumn fits with both explanations because fetuses born at these seasons had more light and warmth during the third trimester of their uterine life than fetuses born during winter or right after.

So why some children become left-handed at five years while the majority become right-handed? Even though the ratio between right- and left-handers was lesser when the father was non-right-handed than when he was right-handed and lesser when the father was non-right-handed and the child was in other presentation than left-cephalic, more than a third of left-handers did not present these factors, and more than half of right-handers presented them. Therefore, more work is needed to find the origin of left-handedness. The hypothesis that we can draw from this study is that with a non-right-handed father and being born in non-left-cephalic presentation, the bias toward right-handedness is weaker and less reinforced during development, thus increasing the likelihood of left-handedness.

Finally, we also looked qualitatively at the six non-lateralized children, to check what could partly explain their absence of lateralization. It seems that all of them had at least one and up to three of these factors associated, significantly or not, with less right-handedness: two of the non-lateralized children had a non-right-handed father; two children were not in left-cephalic presentation, one in breech and the other one in right-cephalic presentation; three of them were born in winter and two were born in spring, and the child born in summer, and for whom we have no data for father’s handedness and side of presentation, was born after only 31 weeks of pregnancy.

These results fit partially with Fagard’s mixed model of handedness [2] which states that hand preference arises from some genetic, probably indirect, influences resulting in a slight advantage for the right side in the vast majority of people, but that self-dynamic, environmental, and cultural factors made this small tendency become a true preference for the right hand during typical development (see also [1,3]). The hypothesis that genetic factors are involved in the right-handedness of typical persons has never been proved by geneticists, despite the fact that many researches were made in that direction (e.g., [83,84]. However, two facts argue for some genetic influence: (1) handedness asymmetries is observed in utero before an effect of environment could intervene, (2) there is an effect of family’s (here father’s) handedness (however, this could be partly due to non-genetic transmission). It has been proposed that postural asymmetry could be the genetic factor indirectly inducing a bias in hand preference [3]. Postural asymmetry could result in a weak manual asymmetry: indeed, the asymmetry is weak at the beginning of life and most children do not have a stable hand preference during the first years [12,13,18,20,21,22], before they engage in the repetitive activity of writing. In addition, adults switch easily hand dominance when they have to, which indicates that hand preference is not as strong as could be thought [85]. Thus, a genetic tendency to favor the right side, possibly of postural origin, is probably reinforced as the fetus explores its environment: the more it uses its right hand and the more it receives feedback and the more it will be likely to use it again, with some kind of snowball effect.

In conclusion, this study shows that, at least in our cohort, father’s handedness and presentation and side of position are associated with the direction of handedness of the child at 5, without explaining fully why about almost 9 out of 10 children become right-handed whereas 1 becomes left-handed. It also shows that number of weeks of pregnancy and season of birth are associated with the degree of handedness.

## Figures and Tables

**Table 1:** Number of children considered for the analyses.

	*n* for Description of the Distribution	*n* for Handedness Analysis
Side position at the 3rd trimester of pregnancy (left/right)	1656	
Side position at the 3rd trimester for cephalic only (left/right)	1475	871
Additional ultrasound recording (side presentation)	444	
Presentation at the third trimester of pregnancy (cephalic/breech)	1819	
At birth: season of birth; number of weeks of gestation	1897	1129
Presentation at birth (cephalic/breech)	1893	
Presentation at birth when identical to presentation at the 3rd trimester (cephalic/breech) (out of 1819 minus 2 missing)	1646	975
Side presentation at the 3rd trimester of pregnancy and presentation at birth (when identical to presentation at the 3rd trimester)	1511	895
Mother’s handedness (R-handed vs. L-handed vs. Ambidextrous; or R-handed vs. NR-handed)	1851	1102
Father’s handedness (idem)	1616	1001
Mother and Father’s handedness (idem)	1575	975
Child’s handedness at 5 (R-handed vs. L-handed vs. NL; or R-handed vs. NR-handed)	1129	

**Table 2:** HI and absHI as a function of season of birth.

	Winter	Spring	Summer	Autumn
HI (SD)	0.67 (0.63)	0.73 (0.57)	0.72 (0.62)	0.74 (0.62)
absHI (SD)	0.91 (0.15)	0.92 (0.13)	0.94 (0.12)	0.96 (0.10)
*n*	256	316	293	264

**Table 3:** Description of the six non-lateralized children in relation to the prenatal factors.

Non-Lateralized Children at 5	Child 1	Child 2	Child 3	Child 4	Child 5	Child 6	% of NL Children Showing the Factor (vs. % of RH and LH Children Showing the Factor)
Father’s handedness	RH (1)	RH (1)	Ambidextrous (0.23)	Left-handed (−0.54)	RH (1)	unknown	(Non-RH father) 40% (11.7%; 19.4%)
Birth presentation	Cephalic	Cephalic	Breech	Cephalic	Cephalic	unknown	(Breech) 20% (3.4%; 5.2%)
Birth presentation and side	Cephalic left	Cephalic right	Breech	Cephalic left	Cephalic left	unknown	(other than left-cephalic) 40% (42.1%; 49%)
Saison of birth	Spring	Winter	Spring	Winter	Winter	Summer	(Winter or spring) 83.3% (50.6%; 49.6%)
Number of weeks of gestation	39	39	39	38	38	31	(Mean n of weeks) 37.3 (39.2; 39.3)
Very premature	No	No	No	No	No	Yes	(very premature) 16.7% (1%; 1.5%)
Mother (IL)	RH (1)	RH (0.92)	RH (1)	RH (1)	LH (−1)	RH (0.83)	(Non-RH mother) 16.7% (9.7%; 9.9%)
Sex	boy	boy	girl	girl	boy	girl	(% of boys) 50% (53%; 56%)
HI	0.13	0.25	0	0.25	0	−0.25	(Mean HI) 0.06 (0.94; −0.90)

## Data Availability

The data that support the findings of this study are available upon request from the EDEN steering committee. Readers may contact etude.eden@inserm.fr to request the data.
